# Using discrete choice experiments to inform the design of complex interventions

**DOI:** 10.1186/s13063-019-3186-x

**Published:** 2019-03-04

**Authors:** Fern Terris-Prestholt, Nyasule Neke, Jonathan M. Grund, Marya Plotkin, Evodius Kuringe, Haika Osaki, Jason J. Ong, Joseph D. Tucker, Gerry Mshana, Hally Mahler, Helen A. Weiss, Mwita Wambura, The VMMC study team

**Affiliations:** 1 Department of Global Health and Development, London School of Hygiene and Tropical Medicine, 15–17 Tavistock Place, London, WC1H 9SH UK; 2 National Institute for Medical Research, PO Box 1462, Mwanza, Tanzania; 3 Centers for Disease Control and Prevention (CDC), Center for Global Health, Division of Global HIV & TB, Atlanta, GA 30333 USA; 4 Jhpiego, 1615 Thames Street, Baltimore, MD 21231 USA; 5 Department of Clinical Research, Faculty of Infectious and Tropical Diseases, London School of Hygiene and Tropical Medicine, Kepple Street, London, WC1E 7HT UK; 6 University of North Carolina Chapel Hill Project—China, No. 2, Lujing Road, Guangzhou, 510095 China; 7 FHI 360, 1825 Connecticut Avenue NW, Washington, DC 20009 USA; 8 MRC Tropical Epidemiology Group, Department of Infectious Disease Epidemiology, London School of Hygiene and Tropical Medicine, Kepple Street, London, WC1E 7HT UK

**Keywords:** Choice experiment, Preferences, HIV, Tanzania, Voluntary medical male circumcision, Formative research

## Abstract

**Background:**

Complex health interventions must incorporate user preferences to maximize their potential effectiveness. Discrete choice experiments (DCEs) quantify the strength of user preferences and identify preference heterogeneity across users. We present the process of using a DCE to supplement conventional qualitative formative research in the design of a demand creation intervention for voluntary medical male circumcision (VMMC) to prevent HIV in Tanzania.

**Methods:**

The VMMC intervention was designed within a 3-month formative phase. In-depth interviews (*n* = 30) and participatory group discussions (*n* = 20) sought to identify broad setting-specific barriers to and facilitators of VMMC among adult men. Qualitative results informed the DCE development, identifying the role of female partners, service providers’ attitudes and social stigma. A DCE among 325 men in Njombe and Tabora, Tanzania, subsequently measured preferences for modifiable VMMC service characteristics. The final VMMC demand creation intervention design drew jointly on the qualitative and DCE findings.

**Results:**

While the qualitative research informed the community mobilization intervention, the DCE guided the specific VMMC service configuration. The significant positive utilities (*u*) for availability of partner counselling (*u* = 0.43, *p* < 0.01) and age-separated waiting areas (*u* = 0.21, *p* < 0.05) led to the provision of community information booths for partners and provision of age-separated waiting areas. The strong disutility of female healthcare providers (*u* = − 0.24, *p* < 0.01) led to re-training all providers on client-friendliness.

**Conclusion:**

This is, to our knowledge, the first study documenting how user preferences from DCEs can directly inform the design of a complex intervention. The use of DCEs as formative research may help increase user uptake and adherence to complex interventions.

**Electronic supplementary material:**

The online version of this article (10.1186/s13063-019-3186-x) contains supplementary material, which is available to authorized users.

## Background

User social and behavioural characteristics can attenuate the effectiveness of complex interventions, which exacerbates the challenge of testing their effectiveness in trials. For example, use of antiretroviral (ART)-based vaginal and oral pre-exposure prophylaxis (PrEP) was effective in five of eight randomized controlled trials (RCTs) [1–8], with the differing results largely attributed to sub-optimal adherence rather than a lack of product efficacy [9, 10]. Incorporating user preferences at the intervention design phase may be useful for enhancing the uptake of and adherence to complex health interventions.

Although there is a growing literature reporting on the formative research processes applied in intervention development [11–15], to date only few studies or theoretical frameworks provide methodological guidance on conducting such formative research [16–18]. Qualitative research can identify a range of barriers and facilitators of accessing services, and participatory rating or ranking exercises can retrieve ordinal measures of importance. However, when faced with numerous service characteristics and a need to prioritize between them, neither of these approaches provides concrete guidance for implementers on which combination of characteristics will achieve the greatest uptake. Some studies have used discrete choice experiments (DCEs) to inform programme design and identify potential barriers and facilitators of uptake [19–21]. DCEs are a survey-based approach to eliciting user preferences. They allow the estimation of user values in the absence of observable markets, where services are provided for free or have not yet been introduced. They can measure the strength of preferences between service attributes, for example, valuing waiting times, prices and provider gender, independently. Lastly, they can identify where preferences differ between individuals, which is particularly useful when complex interventions include targeting specific user groups.

**Fig. 1:**
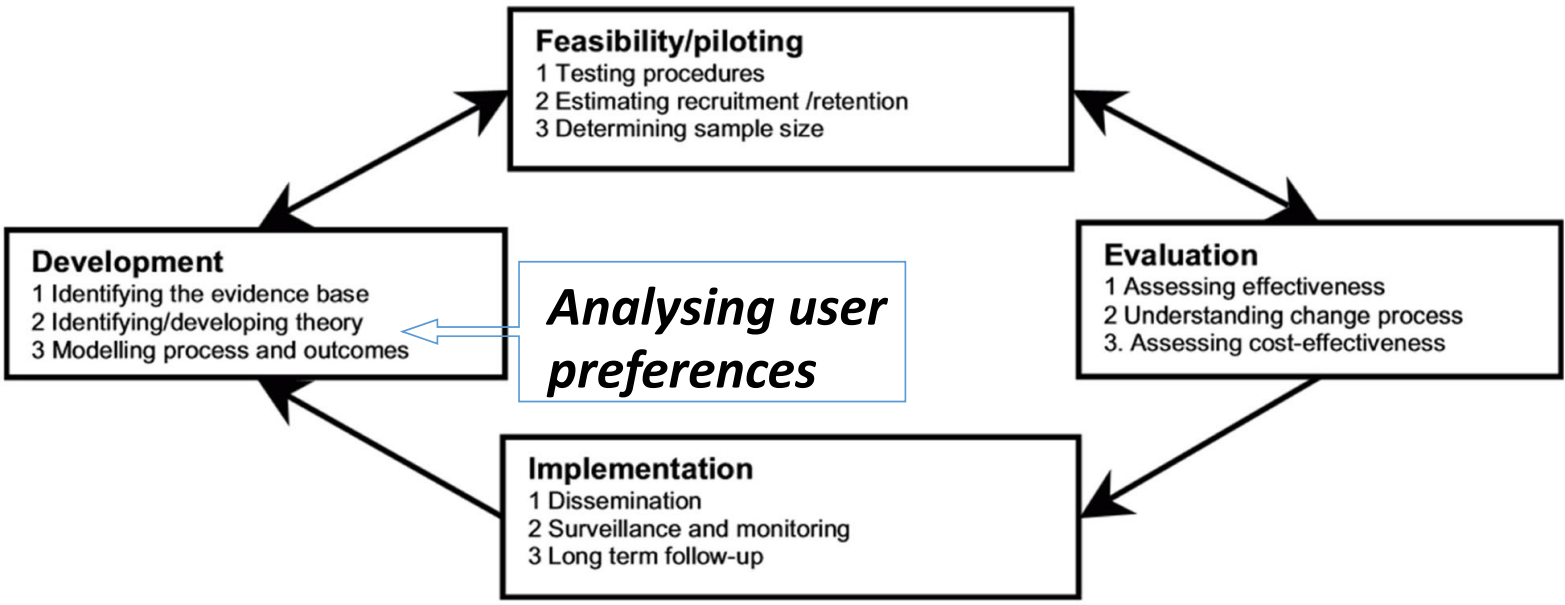
Key elements of the development and evaluation process. Source: adapted from Medical Research Council guidelines [36]

The purpose of this study is to describe how the DCE methodology can be used to inform the development of complex interventions. To demonstrate the approach, we use the case of an intervention to increase uptake of voluntary medical male circumcision (VMMC) among adult men in Tanzania (Table 1), for which the DCE was integral in prioritizing the intervention components. We argue that this may be a useful addition to the current MRC guidelines for developing and evaluating complex interventions [21], as proposed in Fig. 1.

**Table 1:** Voluntary medical male circumcision demand creation intervention in Tanzania

Following strong evidence for the effectiveness of voluntary medical male circumcision (VMMC) in reducing men’s risk of acquiring HIV, Tanzania was identified as one of 14 high-priority countries for VMMC scale-up [37]. Tanzania, which started rolling out VMMC in 2009, has a national adult HIV prevalence of 5.0% [38] and an adult male circumcision prevalence of 72% [39, 40]. The Tanzanian HIV and AIDS Strategic Plan prioritized scaling-up VMMC to males aged 10–34 years in eight regions (which was later increased to 12 regions due to administrative divisions), including Njombe and Tabora [41]. The National AIDS Control Programme established a target of circumcising 2.2 million eligible males aged 10–34 years in these 12 regions [40]. Significant progress has been made towards these targets: from 2009 to 2015, almost 1.2 million VMMCs were performed in the priority regions [42].	
VMMC in Tanzania has predominantly attracted a young male population: As of 2012, 82% of VMMC clients in Njombe and Iringa were adolecent boys between 10-19 years old [43]. For a more immediate impact on HIV incidence, reaching an older, sexually active but not yet HIV-infected client base would be preferable. Social barriers in relation to adult men accessing VMMC services have been documented in Tanzania [44, 45]. To evaluate a model of demand creation and service delivery to increase uptake of VMMC among adult men aged ≥ 20 years, a two-phase study was implemented in the Tabora and Njombe regions. Njombe is the region with the highest HIV prevalence at 14.8% [46], and Tabora has a prevalence of 6.4%, which is higher than the national average (5.1%) [47]. In 2012, male circumcision coverage was 49% in Njombe and in Tabora, which is considerably lower than the national average of 72% [47]. Phase 1 of the study consisted of formative research to develop a package of demand creation activities designed to increase the client base of adult men (age 20–34 years).	
Phase 2 was a cluster randomized trial evaluating the impact of the developed complex demand-creation intervention with a goal of increasing the proportion and number of VMMC clients aged 20–34 years whose results are published [33]. In brief, 20 outreach sites in Njombe and Tabora were randomized to receive either a demand-creation intervention package (described in Table 4) plus standard VMMC outreach, or standard VMMC outreach only. The primary outcome was defined as the increase in the proportion of 20–34 year olds among all men being circumcised in the intervention arm. Secondary outcomes compared the total increased uptake compared to the routine VMMC strategy, as well as uptake among all mature men (age ≥ 20 years).	
**Intervention evaluation**	
The RCT showed significant increases in VMMC uptake across men of all ages relative to the control arm (619 versus 393, *p* = 0.03) [33]. In Tabora there was a significant increase in the proportion of men aged 20–34 years (28% in the intervention arm relative to 12% in the control arm; *p* < 0.00), but not in Njombe (11% in the intervention arm and 15% the control arm; *p* = 0.44) where large increases among the younger men were also observed [33]. Further details of the intervention and its evaluation can be found in Wambura et al. [33].

## Methods

We present the mixed-methods formative research in chronological order. First, the conventional formative qualitative research is described. We then present the design of the DCE and how it built on this qualitative work, followed by the methods for DCE data collection and analysis. Finally, we discuss the intervention design features resulting from information generated within the conventional formative research and the contribution of a DCE in this complex intervention.

### Designing an evidence-based demand creation intervention: integrated formative data collection

The DCE was integrated into the qualitative data collection activities.

Table 2 presents an overview of the fieldwork process and how the qualitative research and DCEs were conducted in tandem. While the qualitative research team undertook in-depth interviews (IDIs) and participatory group discussions (PGDs), real-time debriefing of qualitative field teams provided qualitative insights for the DCEs. The qualitative team conducted additional iterative cognitive interviews while the DCE tools were developed and subsequently administered the DCE questionnaires. Recruitment for this phase is described in detail in Additional file 1.

**Table 2:** Integrated formative research design

Qualitative research step		Added DCE step
1. Pilot testing in-depth interviews (IDIs) and participatory group discussions (PGDs)		(a) Debriefing qualitative research team; four rounds of pre-pilot individual interviews
	↓	(b) Finalization of pilot tool and experimental design
2. Qualitative research data collection:	↓	(c) DCE pilot interviews (*n* = 50)
Njombe: 10 PGDs and 15 IDIs	↓	(d) Analysis of pilot data to obtain prior utility estimates (priors) used to generate the efficient experimental design
Tabora: 10 PGDs and 15 IDIs		
		(e) DCE survey:
		Njombe (*n*=159); Tabora (*n*=166)
3. Intervention design		(f) Present and interpret qualitative and DCE results to tailor final VMMC demand creation intervention

*DCE* discrete choice experiment, *VMMC* voluntary medical male circumcision

### Conventional formative research

Following a scoping literature review around behaviour change and VMMC, the information–motivation–behavioural (IMB) theory was chosen as the most suitable behaviour change framework [22]. Gaps were identified and formative research underpinning the IMB theory was conducted to identify individual and community-level factors that facilitate or limit the uptake of VMMC in each of the three constructs. Findings from this formative research were used to develop the intervention that was evaluated in phase 2 of the study and have been published elsewhere [23]. In brief, the qualitative research team conducted 30 in-depth interviews and 20 participatory group discussions across the two regions, Njombe and Tabora, in rural and urban areas, with women and with younger men (age < 20 years) and older men (age 20 years and older). These interviews aimed to comprehensively understand barriers and facilitators to access VMMC services. When the DCE was added, the scope was expanded to determine which service delivery components to assess within the DCE and thus informed the early stages of the DCE, following the DCE guidelines [24].

### The discrete choice experiment

**Fig. 2:**
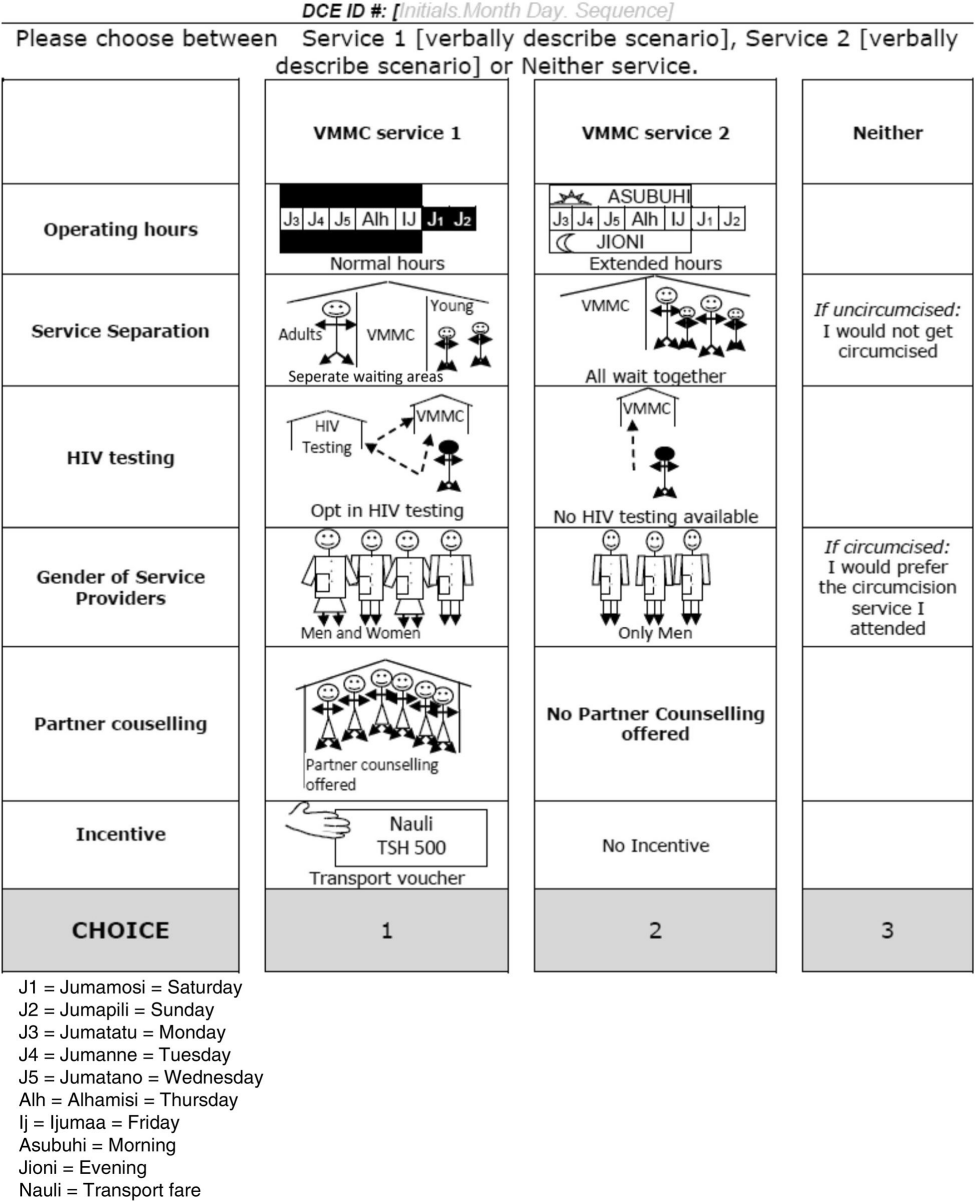
Discrete choice experiment (DCE) choice scenario. VMMC voluntary medical male circumcision

DCEs present respondents with a series of choice scenarios where they are asked to choose between two or more alternative goods or services with simultaneously varying attribute levels (Fig. 2). In making these choices, respondents are forced to trade-off between preferred and less preferred attribute levels presented in each alternative service. Econometric analysis of respondent choices allows one to retrieve the value (utility) derived from each attribute level relative to the others. It can also test whether people with different characteristics, such as age and gender, have significantly different preferences.

Using the estimated utilities of goods (i.e. the sum of the utility of each characteristic), one can estimate relative market shares. If an opt-out alternative is included (i.e. one can choose ‘Neither of the alternatives’), levels of uptake can be predicted. As such, DCEs allow one to explore the impact of varying service characteristics on potential uptake of an intervention in the context of both research and programme implementation [25].

The development of the DCE followed steps as laid out in the standard guidelines [24]. The literature on the uptake of VMMC among adult men (age 20 years and older), including the grey literature of qualitative work from within Tanzania, was reviewed [26, 27]. Following this, the DCE was developed and implemented over a 6-week period of fieldwork (February–March 2014). Potential feasible demand creation strategies were explored using sequential rounds of cognitive interviews and debriefings from two group discussions considering both the key drivers of demand and their pictorial representations.

The final attributes (including pictorial representations) included in the DCE are shown in Fig. 2. The full set of attribute levels are presented in Table 3. The DCE provided participants with a sequence of nine repeated choices between two generic circumcision services and ‘Neither’. For uncircumcised men, ‘Neither’ represented remaining uncircumcised; for circumcised men, ‘Neither’ represented having the same services they had when previously circumcised. Analysis of a pilot DCE survey (*n* = 54) provided the priors to generated a *d*-efficient design using NGENE 1.1 [28]. The DCE design allowed for an analysis capturing the preference heterogeneity for service characteristics by region (Tabora and Njombe), age and circumcision status. Although preferences for financial incentives were also collected, they were not considered an option within this intervention package and were therefore not part of the intervention development process.

**Table 3:** Discrete choice experiment attributes and levels

Attribute	Levels
Time of service	Normal working hours and days
	Extended hours and weekend services
Service separation	Standard service with all clients together
	Separate waiting areas for younger and older men
	Separate services for younger and older men
HIV testing	Opt-out
	Opt-in
	Not available
Gender of service provider	Male and female
	All male
	All female
Female partner counselling	Partner counselling offered in the community
	No partner counselling offered
Incentives	None
	Transport voucher for 500 Tanzanian shillings (TzS)
	Transport voucher for TzS 1500^a^
	Transport voucher for TzS 4500
	Lottery low (1/10 chance of winning TzS 5000)
	Lottery medium (1/10 chance of winning TzS 15,000)
	Lottery high (1/10 chance of winning TzS 45,000)^b^

^a^TzS 500, TzS 1500 and TzS 4500 are roughly equivalent to US$0.30, US$0.90 and US$2.70 in 2015

^b^The expected values of the low and high incentives are equal across the transport voucher and lottery

### Statistical methods

The utility of a given service (VMMC_A_ or VMMC_B_) is estimated as a function of the utilities of the separate service characteristics:

$$ {U}_{\mathrm{VMMC}\ \mathrm{A}},{U}_{\mathrm{VMMC}\ \mathrm{B}}=f\left(\mathrm{time}\ \mathrm{of}\ \mathrm{service}\ \mathrm{provision},\mathrm{service}\ \mathrm{separation},\mathrm{HIV}\ \mathrm{testing},\mathrm{provider}\ \mathrm{gender},\mathrm{partner}\ \mathrm{counselling},\mathrm{financial}\ \mathrm{incentives}\right) $$

$$ {U}_{\mathrm{Neither}}=\mathrm{alternative}\ \mathrm{specific}\ \mathrm{constant}\ {\left(\mathrm{ASC}\right)}_{\mathrm{Neither}}+\mathrm{own}\ \mathrm{circumcision}\ \mathrm{status} $$

These service characteristics varied in each choice scenario, as determined in the experimental design. Respondents could also choose to not be circumcised in either VMMC_A_ or VMMC_B_ and then choose ‘Neither’, which in the first instance is given an alternative specific constant (ASC). To obtain total utility for a given service, one can sum the utility values of the specified attribute levels.

Service attributes and respondent covariates allow for exploration of observed preference heterogeneity and will be indicated as the multinomial logit model with interactions (MNLX). In this case we allowed for variation by respondents’ region (Tabora/Njombe, with _NJO indicating variation in preferences of those living in Njombe relative to Tabora) and age (< 21 years, ≥ 21 years).^1^ Categorical variables were coded using effects coding (e.g. 1 and − 1 for a dichotomous variable), which imposes a central utility of 0; thus, the utilities represent *relative* preferences [29]. The magnitude of the utilities themselves has no meaning if not interpreted relative to the magnitude of utilities of other attributes. The significance levels of the utilities test whether the utilities are significantly different from the utility of the reference (i.e. the omitted) category.

Using the multinomial logit model (MNL) and to allow for unobserved preference heterogeneity and explore scale heterogeneity, we applied the generalized multinomial logit model (GMXL) estimator [30]. The gamma coefficient tests for unobserved preference heterogeneity and the tau coefficient tests for scale heterogeneity. If there is no scale heterogeneity, then the model can be collapsed to a random parameters logit model. Where there is neither scale nor unobserved heterogeneity, the model can be collapsed to the multinomial logit model. Estimation was undertaken using NLOGIT 5.0 [31]. The GMXL model parameters were obtained by simulation; 500 iterations using Halton draws were applied. Service configuration attribute parameters were specified as random with normal distributions. The incentives (lottery and transport reimbursement) were specified with a triangular distribution, as is common around price attributes to impose a minimum of 0 [32].

### Intervention design

Following the qualitative and DCE analyses, a teleconference was held with the full project team to review the findings and revise the initial intervention design for implementation and to evaluate the tailored intervention with routine demand creation for VMMC within a cluster randomized controlled trial (CRT). This CRT is described in detail by Wambura et al. [33].

## Results

### DCE sample description and analysis

The fieldwork team administered 325 DCE surveys, 159 in Njombe and 166 in Tabora, with approximately equal numbers in urban and rural areas. Participants’ background characteristics are presented in Additional file 2. To sum, Christianity is the main religion among participants from Njombe (96%), with participants from Tabora having more diverse religious backgrounds: 51% Christian, 24% Muslim and 25% other religions. Ethnic composition was different between the regions: Bena participants were the majority in Njombe (83%), while in Tabora participants were primarily Sukuma (55%) and Nyamwezi (36%). Education and wealth (based on mobile phone and land ownership) appear similar across regions. Sexual behaviour appears more risky among participants from Tabora than from Njombe, with 51.3% and 38.6% (*p* ≤ 0.01), respectively, reporting more than one partner in the past year and 38.6% versus 50.4% (*p* < 0.01), respectively, reported condom use at last sex act. Only a third of participants self-reported not being circumcised, with similar rates across the regions. Knowledge and attitudes around VMMC also appear relatively similar across regions.

**Fig. 3:**
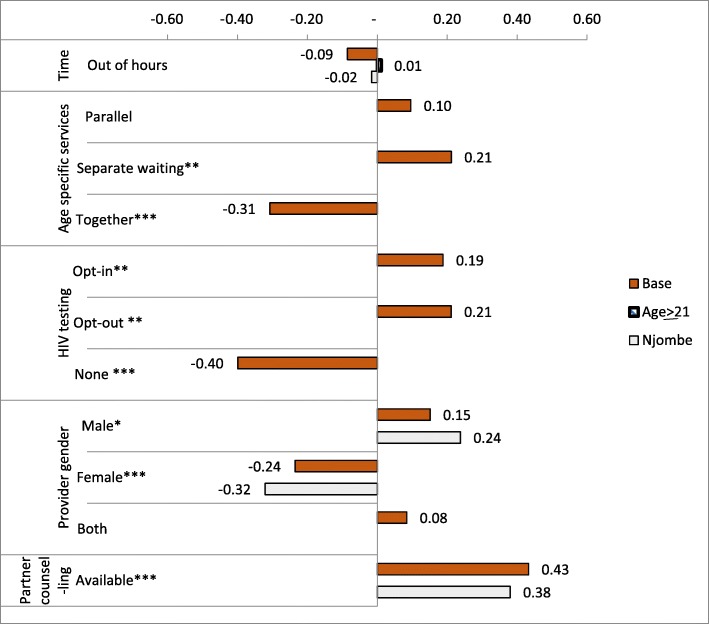
Relative strength of preferences for voluntary medical male circumcision service attributes and their variation by region and circumcision status. Dark bars represent the base utility of the attribute. If preferences are significantly different between groups of respondents, these are shown below the base utility bars for significant variation by region (light bar showing utility values in Njombe) and by age (striped bar showing utility values where those aged 21 years or older had significantly different preferences from those aged 18–20 years). *: *p* ≤ 0.1, **: *p* < 0.05, ***: *p* < 0.01

Full estimation results are presented in Additional file 3; it can be seen that relative utilities were largely robust across estimators. From here onwards we focus on the estimated utilities generated from the MNLX estimator, which estimates the main effects (i.e. those of the service attributes) and tests for preference heterogeneity by region and age, our key interest. Figure 3 shows the relative strength of preferences and the significant differences in preferences across regions and circumcision status. Where preferences did not significantly differ between region or circumcision status, they are not shown, but full utility estimates are presented in Additional file 3.

On average, availability of VMMC services outside standard working hours was not a significant driver of choice. All men preferred to have waiting areas that were separated by age (*u* = 0.21, *p* < 0.05). There was a relatively strong dislike for removing HIV testing from the VMMC process (*u* = − 0.40, *p* < 0.01). The service characteristic with the greatest utility was partner counselling (*u* = 0.43, *p* < 0.01), informing female partners of the need for post-operative abstinence and wound care, and was strongest among men in Tabora (*u* = 0.47, *p* < 0.05). Male-only service providers were preferred (*u* = 0.15, *p* < 0.1), with men in Njombe having four times stronger preferences for all-male service providers than men in Tabora (*u*_*Njombe*_ = 0.15 + 0.09 = 0.24; *u*_*Tabora*_ = 0.151–0.09 = 0.06).

### The final intervention design

Following analysis of the formative research, results from both the DCE and the qualitative study were presented to the full project team for translation into a research design. The qualitative research largely informed community mobilization messaging, and the DCE largely informed the VMMC service delivery model in the intervention arm.

### DCE influence on intervention design

#### Time of service

Previously, it was known that mature men had challenges with fitting in VMMC alongside their livelihoods. It was thought that providing weekend or evening services may alleviate the latter. The DCE shows that this was unlikely to be a driving factor. During the campaign, communities were informed that evening VMMC services were available upon request, but evening services were otherwise not routinely provided.

#### Service separation

The strong and positive preference for a VMMC service providing separated waiting areas prompted setting up separate waiting areas for adolescents and adult men, particularly ensuring that the waiting areas were out of view of each other. It has not been previously recognized how strongly men of all ages felt about their privacy relative to other VMMC clients.

#### HIV testing

Some men had expressed reluctance to present for VMMC due to the HIV testing requirement. The positive value men place on the availability of HIV testing suggested that HIV testing was valued, and led the programme to maintain HIV testing as part of routine client flow and include it in the provider training to re-emphasize its voluntary nature to clients. If strong preferences had been identified against testing, alternative client flows would have been explored, where testing would not be the default.

#### Gender of service provider

The DCE highlighted how strongly uncomfortable men felt with female staff. It was not feasible to increase male providers. The mobile VMMC service relied on the public health system’s supply of healthcare workers and was unable to influence the gender of providers supplied. However, re-training of providers emphasized the importance of professionalism in VMMC provision, and this was relayed in the community campaign.

#### Partner counselling

The qualitative research identified women as important influences on men’s circumcision decisions [23]. The DCE pre-evaluated one option of addressing this: providing counselling and information to partners about the importance of post-natal abstinence and post-operative care. This was highly valued in the DCE and then implemented by setting up information booths in the community for women.

#### Intervention and evaluation

Wambura et al. [33] summarize the new intervention and the status quo/control arm against which it was evaluated, highlighting how the findings from the DCE fed into the intervention arm services (Table 4). The new complex intervention focused on targeted demand creation among adult men (age 20–34 years) with a mass-media campaign and reconfigured services. Using trucks with mega-phones in the communities, radio spots, flyers and posters, and local community peer educators, communities received the key intervention messages. The community was informed about the highly trained staff, addressing issues related to perceptions around female staff and professional staff, and the availability of community-based information booths for female partners. Local peer educators also encouraged men to present for VMMC. Partner counselling booths were set up in the community where women could get information on post-operative abstinence and care. While not operationally feasible to engage all male service providers, re-training was undertaken to ensure providers behaved sensitively towards clients and to emphasize the optional nature of HIV testing prior to VMMC. Separate waiting areas were provided.

**Table 4:** Overview of the routine VMMC service (control arm) and the complex intervention arm as characterized and influenced by the DCE findings, by DCE attribute

Attribute^a^	Key DCE finding	Control	VMMC service intervention (approach to address issue)	Community complement
Out-of-hours service	Not significant	No	Upon request	
Service separation by age	Age separated preferred	Joint waiting areas	Separate waiting areas	
Partner counselling	Available preferred	Not available	Available	Female-friendly information booths placed in community
HIV testing	Available preferred	Opt-out	Opt-out (staff re-training to emphasize HIV testing not required)	Reiteration of HIV testing not required for VMMC in messaging
Service provider genders	Male providers preferred	Both male and female	Both male and female (staff re-training for client friendliness)	Media emphasised staff professionalism

*DCE* discrete choice experiment, *VMMC* voluntary medical male circumcision

^a^Incentives were not considered as part of this intervention and were purely included in the DCE for exploratory analysis

## Discussion

This paper presents how DCEs can complement conventional qualitative research to prioritize service components most critical to the target population of adult men in Tanzania. The DCE results led to the inclusion of two key service components. Firstly, the strong preferences among both the younger men and the older men revealed the importance of age-separated waiting areas at the VMMC facilities. Waiting areas for VMMC were rearranged to allow for younger and older men to wait separately and out of view of each other. Secondly, the strong preference for partner counselling led to the introduction of partner counselling booths, staffed by both female health workers and female community peers who provided one-on-one and small group information for female partners about circumcision care and post-circumcision abstinence. Out-of-hours service provision was preferred in Njombe, but the utility was relatively small compared with the value placed on separated waiting areas and partner counselling service. This allowed the intervention to focus less effort on promoting out-of-hours VMMC services, although they remained available upon request. The suggested preference for opt-out testing led to an intensification of the information campaign, ensuring clients were comfortable requesting not to be tested for HIV and staff were re-trained to emphasize the right to opt-out of HIV testing to clients. Officially, however, both the intervention and the control arms maintained a policy of opt-out HIV testing.

DCEs have great potential to inform the design of complex interventions. DCEs can indicate the relative strength of preferences and identify differences in preferences between population groups, which suggest relative differences in uptake by composition of the intervention package. This paper adds to the literature on methods for the formative phase of complex interventions. The DCE’s contribution is in estimating the relative strength of preferences for intervention components, where DCEs can help distinguish the relative importance to users of various intervention elements that had previously been combined as a package. In doing so, it can inform the optimal composition of interventions to maximize uptake.

The development of this VMMC intervention was informed both by traditional qualitative research and complemented by a DCE. Qualitative research led to the development of targeted messages and appropriate community engagement approaches, while the DCE identified priority service attributes for inclusion and highlighted, in particular, the importance adult men placed on separate waiting areas for adolescent and adult males.

The integrated approach to DCE implementation had a few limitations. As the DCE had to fit into the pre-established formative phase timelines, it did not allow for theory-based qualitative analysis to be finalized to inform the DCE design. In this case, there was a reasonably large qualitative literature on men’s preferences for VMMC in Tanzania; thus, this is unlikely to have undermined the identification of appropriate attributes and levels. In future applications, it is recommended to allow for additional time between the qualitative formative work and the DCE implementation to ensure the DCE is fully informed by robust qualitative analysis. In retrospect, sampling of younger men would have augmented the understanding of how preferences may be different between adolescent men and adult men. This was recognized, but including men younger than 18 years old would have required parental consent, and was considered not feasible within the formative research timeline. Although our main objective was to increase uptake among mature men, it appears that the service configuration changes, like separate waiting areas, were preferred both by younger and older men, as suggested by the large increases in uptake among all age groups. Lastly, the decision to be circumcised is only relevant to uncircumcised men, but to recruit only uncircumcised men would have added costs and time, potentially introducing response bias. Nevertheless, our results appear to have informed the development of a highly effective intervention. However, uptake is only one part of intervention effectiveness, and the ultimate intervention impact is an interaction between efficacy and use. To model intervention impact using DCE uptake predictions, as proposed by Terris-Prestholt et al. [34], HIV risk profiles should be collected and sub-analysis by risk profile undertaken; this could only be done with more robust sample sizes.

There are a number of feasibility issues related to incorporating DCEs into the design phase of pragmatic trials and complex interventions. Firstly, the time required to collect and analyse DCE data can be long, limiting the extent to which this method can rapidly inform interventions. Secondly, DCEs require funding in addition to the primary research study, which may be difficult to justify without providing evidence of its usefulness in modifying the design of the intervention to maximize uptake. Although in this case study the programmers found that the DCE provided concrete design recommendations, how strongly different the intervention would otherwise have been with solely qualitative formative research, or the impact of it on the uptake, would need to be tested. This could follow a research design similar to that by Tang et al. which tested the best crowd-sourced HIV testing promotional videos against the conventionally used promotional video [35]. They found crowd-sourcing a cheaper and no less effective approach to designing these demand creation videos. Similarly, one might test the impact of a DCE-informed demand creation intervention against one developed solely based on qualitative formative research, and compare the costs as well as the uptake across the intervention and control arms.

Although all relevant drivers of demand must be included in DCE scenarios, some of these may not be modifiable for pragmatic reasons. Measuring preferences for non-modifiable attributes may still be critical for identifying key bottlenecks to use and for longer-term health system planning, although they may not be useful for designing shorter-term interventions within health system constraints. For example, ‘All male service providers’ was identified as very important but was not feasible in the short term due to structural shortages of male service providers. However, identification of important but unchangeable service characteristics can help interpret results in cases where a trial shows no impact, and are recommended to still be included in the DCE.

This study suggests two key areas for future research. Firstly, although this study did not include a process evaluation to better understand how the intervention components affected uptake during the trial, we do recommend this for future studies to further develop the use of DCEs for intervention design and to test the validity of the DCE predictions. Secondly, we need to estimate the cost-effectiveness of DCEs in complex intervention design. If trials fail to show the effectiveness of efficacious new technologies due to intervention delivery approaches, up-front investment in the delivery intervention could be a highly efficient use of resources. One approach to investigating whether uptake could significantly change an intervention’s impact would be to apply a value-of-information analysis in the early intervention development stage. This models how important the uptake parameter is in an intervention’s cost-effectiveness and how likely it would be in changing resource allocation decisions. If uptake is found critical, then a DCE may be more worth undertaking prior to complex intervention trials. An additional logical extension would be to use the DCE estimates to model the changes in uptake caused by modifying services. Task-shifting activities to lower healthcare worker grades who may technically perform the procedure equally well, for example, may appear as an appropriate cost saver, but the impact on the uptake is often unknown. DCEs can provide insights into the implications of changes, and when coupled with service component costs can look at the incremental cost-effectiveness of varying service components [34].

## Conclusion

This paper has shown how DCEs may have ultimately contributed to a more effective intervention design. DCEs provide an important complement to the methodologies for developing complex interventions. For this application, the relative strength of preferences contributed to prioritization of the components of the complex intervention. DCEs can provide quantitative guidance on how to maximize uptake of interventions, in both simple and complex interventions, and may improve the quality of intervention research and implementation.

## Additional files

Additional file 1: Detailed sampling procedures for the integrated formative research (DOCX 14 kb)

Additional file 2: Sample overview. **Table S1.** Sample descriptive statistics, by region (DOCX 27 kb)

Additional file 3: Comparison of estimators. **Table S2.** Full model estimates. **Figure S1** Comparison of main effects by estimator (DOCX 164 kb)

## Abbreviations

CRT: Cluster randomized trial
DCE: Discrete choice experiment
GMXL: Generalized mixed logit model
IMB: Information–motivation–behavioural
RCT: Randomized control trial
VMMC: Voluntary medical male circumcision

## Footnotes

1 Although the target population is men 20–24 years of age, our sample of 18 and 19 year olds was too small to analyse independently. To allow analysis of younger versus older men, we categorized men aged 18–21 years as the younger age group, and age 21 years and above as older men, despite this not strictly conforming to the CRT age categories.

## References

[1] Rees H, Delany-Moretlwe S, Baron D, Lombard C, Gray G, Myer L, Panchia R, Schwartz J, Doncel G. FACTS 001 Phase III Trial of Pericoital Tenofovir 1% Gel for HIV Prevention in Women. Seattle: CROI; 2015.

[2] Choopanya K, Martin M, Suntharasamai P, Sangkum U, Mock PA, Leethochawalit M, Chiamwongpaet S, Kitisin P, Natrujirote P, Kittimunkong S, et al. Antiretroviral prophylaxis for HIV infection in injecting drug users in Bangkok, Thailand (the Bangkok Tenofovir Study): a randomised, double-blind, placebo-controlled phase 3 trial. Lancet. 381(9883):2083–90. 10.1016/S0140-6736(13)61127-7 23769234

[3] Baeten JM, Donnell D, Ndase P, Mugo NR, Campbell JD, Wangisi J, Tappero JW, Bukusi EA, Cohen CR, Katabira E (2012). Antiretroviral prophylaxis for HIV prevention in heterosexual men and women. N Engl J Med.

[4] Grant RM, Lama JR, Anderson PL, McMahan V, Liu AY, Vargas L, Goicochea P, Casapía M, Guanira-Carranza JV, Ramirez-Cardich ME (2010). Preexposure chemoprophylaxis for HIV prevention in men who have sex with men. N Engl J Med.

[5] Marrazzo JM, Ramjee G, Richardson BA, Gomez K, Mgodi N, Nair G, Palanee T, Nakabiito C, van der Straten A, Noguchi L (2015). Tenofovir-based preexposure prophylaxis for HIV infection among African women. N Engl J Med.

[6] Thigpen MC, Kebaabetswe PM, Paxton LA, Smith DK, Rose CE, Segolodi TM, Henderson FL, Pathak SR, Soud FA, Chillag KL (2012). Antiretroviral preexposure prophylaxis for heterosexual HIV transmission in Botswana. N Engl J Med.

[7] Van Damme L, Corneli A, Ahmed K, Agot K, Lombaard J, Kapiga S, Malahleha M, Owino F, Manongi R, Onyango J (2012). Preexposure prophylaxis for HIV infection among African women. N Engl J Med.

[8] Abdool Karim Q, Abdool Karim SS, Frohlich JA, Grobler AC, Baxter C, Mansoor LE, Kharsany AB, Sibeko S, Mlisana KP, Omar Z (2010). Effectiveness and safety of tenofovir gel, an antiretroviral microbicide, for the prevention of HIV infection in women. Science.

[9] van der Straten A, Van Damme L, Haberer JE, Bangsberg DR (2012). Unraveling the divergent results of pre-exposure prophylaxis trials for HIV prevention. AIDS.

[10] Saag MS (2015). Preventing HIV in women—still trying to find their VOICE. N Engl J Med.

[11] Vastine A, Gittelsohn J, Ethelbah B, Anliker J, Caballero B (2005). Formative research and stakeholder participation in intervention development. Am J Health Behav.

[12] Silk KJ, Bigbsy E, Volkman J, Kingsley C, Atkin C, Ferrara M, Goins LA (2006). Formative research on adolescent and adult perceptions of risk factors for breast cancer. Soc Sci Med.

[13] Mbonye AK, Magnussen P, Chandler CI, Hansen KS, Lal S, Cundill B, Lynch CA, Clarke SE (2014). Introducing rapid diagnostic tests for malaria into drug shops in Uganda: design and implementation of a cluster randomized trial. Trials.

[14] Sureshkumar K, Murthy GV, Kinra S, Goenka S, Kuper H (2015). Development and evaluation of a smartphone-enabled, caregiver-supported educational intervention for management of physical disabilities following stroke in India: protocol for a formative research study. BMJ Innov.

[15] Greenland K, Chipungu J, Chilengi R, Curtis V (2016). Theory-based formative research on oral rehydration salts and zinc use in Lusaka, Zambia. BMC Public Health.

[16] Chandler CI, Burchett H, Boyle L, Achonduh O, Mbonye AK, Diliberto D, Reyburn H, Onwujekwe O. Examining intervention design: lessons from the development of eight related malaria health care intervention studies. Health Syst Reform. 2016;2(4):373-88. 10.1080/23288604.2016.1179086 PMC6176770 31514719

[17] De Silva MJ, Breuer E, Lee L, Asher L, Chowdhary N, Lund C, Patel V (2014). Theory of Change: a theory-driven approach to enhance the Medical Research Council's framework for complex interventions. Trials.

[18] Power R, Langhaug LF, Nyamurera T, Wilson D, Bassett MT, Cowan FM (2004). Developing complex interventions for rigorous evaluation—a case study from rural Zimbabwe. Health Educ Res.

[19] Kruk ME, Paczkowski M, Mbaruku G, de Pinho H, Galea S (2009). Women's preferences for place of delivery in rural Tanzania: a population-based discrete choice experiment. Am J Public Health.

[20] Struik MH, Koster F, Schuit AJ, Nugteren R, Veldwijk J, Lambooij MS (2014). The preferences of users of electronic medical records in hospitals: quantifying the relative importance of barriers and facilitators of an innovation. Implement Sci.

[21] van Helvoort-Postulart D, van der Weijden T, Dellaert BG, de Kok M, von Meyenfeldt MF, Dirksen CD (2009). Investigating the complementary value of discrete choice experiments for the evaluation of barriers and facilitators in implementation research: a questionnaire survey. Implement Sci.

[22] Cornman DH, Schmiege SJ, Bryan A, Benziger TJ, Fisher JD (2007). An information-motivation-behavioral skills (IMB) model-based HIV prevention intervention for truck drivers in India. Soc Sci Med.

[23] Osaki H, Mshana G, Wambura M, Grund J, Neke N, Kuringe E, Plotkin M, Mahler H, Terris-Prestholt F, Weiss H (2015). "If you are not circumcised, I cannot say yes": the role of women in promoting the uptake of voluntary medical male circumcision in Tanzania. PLoS One.

[24] Mangham LJ, Hanson K, McPake B (2009). How to do (or not to do) … Designing a discrete choice experiment for application in a low-income country. Health Policy Plan.

[25] de Bekker-Grob EW, Ryan M, Gerard K (2012). Discrete choice experiments in health economics: a review of the literature. Health Econ.

[26] Plotkin M, Mziray H, Kuver J, Prince J, Curran K, Mahler H. A qualitative assessment of views and preferences concerning voluntary medical male circumcision in Iringa Region, Tanzania: USAID, MCHIP; 2011. https://www.malecircumcision.org/resource/qualitative-assessment-views-and-preferences-concerning-voluntary-medical-male-circumcision. Accessed 28 Jan 2019.

[27] Wambura M, Mwanga J, Mosha J, Mshana G, Mosha F, Changalucha J (2009). Situation analysis for male circumcision in Tanzania.

[28] Ngene 1.1.1 User Manual & Reference Guide, Version 16 February 2012, Sydney.

[29] Bech M, Gyrd-Hansen D (2005). Effects coding in discrete choice experiments. Health Econ.

[30] Fiebig D, Keane MP, Louvier J, Wasi N (2010). The generalized multinomial logit model: accounting for scale and coefficient heterogeneity. Mark Sci.

[31] Greene B. NLOGIT 5.0. Plainview, NY: Econometric Software, Inc.; 2012.

[32] Michaels-Igbokwe C, Terris-Prestholt F, Lagarde M, Chipeta E, Cairns J (2015). Young people’s preferences for family planning service providers in rural Malawi: a discrete choice experiment. PLoS One.

[33] Wambura M, Mahler H, Grund JM, Larke N, Mshana G, Kuringe E, Plotkin M, Lija G, Makokha M, Terris-Prestholt F (2017). Increasing voluntary medical male circumcision uptake among adult men in Tanzania. AIDS.

[34] Terris-Prestholt F, Quaife M, Vickerman P (2016). Parameterising user uptake in economic evaluations: the role of discrete choice experiments. Health Econ.

[35] Tang W, Han L, Best J, Zhang Y, Mollan K, Kim J, Liu F, Hudgens M, Bayus B, Terris-Prestholt F (2016). Crowdsourcing HIV test promotion videos: a noninferiority randomized controlled trial in China. Clin Infect Dis.

[36] Medical Research Council (2008). Developing and evaluating complex interventions: new guidance.

[37] Njeuhmeli E, Forsythe S, Reed J, Opuni M, Bollinger L, Heard N, Castor D, Stover J, Farley T, Menon V (2011). Voluntary medical male circumcision: modeling the impact and cost of expanding male circumcision for HIV prevention in eastern and southern Africa. PLoS Med.

[38] Tanzania country profile. http://www.unaids.org/en/regionscountries/countries/unitedrepublicoftanzania. Accessed 11 July 2015.

[39] Tanzania Commission for AIDS (TACAIDS) ZACZ, National Bureau of Statistics (NBS), Office of the Chief Government Statistician (OCGS), and ICF International (2013). Tanzania HIV/AIDS and Malaria Indicator Survey 2011/12: key findings.

[40] Ministry of Health and Social Welfare URoT. National strategy for scaling-up male circumcision for HIV prevention: enhancing men’s role in HIV prevention. Dar es Salaam: National AIDS Control Programme; 2010.

[41] Program NAC, Ministry of Health and Social Welfare URoT (2013). Third Health Sector HIV and AIDS Strategic Plan (HSHSP III) 2013–2017.

[42] WHO Progress Brief: voluntary medical male circumcision for HIV prevention in 14 priority countries in East and Southern Africa. http://apps.who.int/iris/bitstream/10665/179933/1/WHO_HIV_2015.21_eng.pdf?ua=1. Accessed 9 Sept 2015.

[43] Ashengo TA, Hatzold K, Mahler H, et al. Voluntary medical male circumcision (VMMC) in Tanzania and Zimbabwe: service delivery intensity and modality and their influence on the age of clients. PLoS One. 2014;9(5):e83642. 10.1371/journal.pone.0083642 PMC4011872 24801882

[44] Plotkin M, Castor D, Mziray H, Kuver J, Mpuya E, Luvanda PJ, Hellar A, Curran K, Lukobo-Durell M, Ashengo TA (2013). "Man, what took you so long?" Social and individual factors affecting adult attendance at voluntary medical male circumcision services in Tanzania. Glob Health Sci Pract.

[45] Herman-Roloff A, Llewellyn E, Obiero W, Agot K, Ndinya-Achola J, Muraguri N, Bailey RC (2011). Implementing voluntary medical male circumcision for HIV prevention in Nyanza Province, Kenya: lessons learned during the first year. PLoS One.

[46] Results from the 2011 12 Tanzania HIV/AIDS and Malaria Indicator Survey. https://nbs.go.tz/nbs/takwimu/this2012/HIVFactsheetbyRegion.pdf. Accessed 11 July 2015.

[47] Ministry of Health and Social Welfare/National AIDS Control Program (2014). Voluntary Medical Male Circumcision Country Operational Plan. 2014–2017.

