# Supplementary material for: Using discrete choice experiments to inform the design of complex interventions
Source: Trials. 2019 Mar 4;20:157. doi: 10.1186/s13063-019-3186-x (PMC6399844; doi:10.1186/s13063-019-3186-x)
Supplement: Supplementary file 1 — Detailed sampling procedures for the integrated formative research (DOCX 14 kb) [file 13063_2019_3186_MOESM1_ESM.docx]

# Additional File 1: Detailed sampling procedures for the integrated formative research

A multistage cluster sampling design was used. At the first stage, a list of all districts in the region that had received VMMC outreach services from MCHIP in the last 12 months was generated. One district from each region was selected purposively into the study. In each selected district a list of all sites that had offered VMMC outreach campaign in the last twelve months was generated. The sites were stratified by residence (rural, urban).

In the second stage, one rural site and one urban site were randomly selected from each stratum to participate into the study. In the third stage, a list of all sub villages/streets in the selected rural and urban sites was generated. Three sub-villages were selected randomly to participate in the study. The sub-village leaders were then asked to develop a list of residents aged 18+ years who were then invited to meet the study team and were consented if willing to participate. Some of these participants took part in the participatory group discussions while others took part in the DCE or both.

The teams also visited the health facility and listed the names and telephone contacts (if available) of all men who were circumcised in the previous seven weeks and aged 20-34 years at the time of circumcision from the VMMC register. The aim was to select circumcised participants for in-depth interviews. From the list, four individuals were randomly selected and were contacted if telephone contacts were available. If they had no telephone contacts, the generated list was stratified by sub-village and village/street. Separate lists were given to the respective sub village/street leaders who were requested to identify those men to be interviewed. Sub village/street leaders identified the men listed and the interviewing team met them privately to ask for permission to take part in the study. If they gave verbal consent, we requested them to give a written consent after having read the informed consent sheet and all their questions responded to their satisfaction. The men who agreed to take part in the study were then used as seeds to bring in other participants who were aged 18+ years for the DCE. All available men aged eighteen years and above who were invited by seeds who were circumcised in the last 7 weeks were also selected to be included in the study. The minimum sample size was estimated to be 320 male.
