# Supplementary material for: Using discrete choice experiments to inform the design of complex interventions
Source: Trials. 2019 Mar 4;20:157. doi: 10.1186/s13063-019-3186-x (PMC6399844; doi:10.1186/s13063-019-3186-x)
Supplement: Supplementary file 2 — Sample overview. Table S1. Sample descriptive statistics, by region (DOCX 27 kb) [file 13063_2019_3186_MOESM2_ESM.docx]

# Additional File 2: Sample overview

Table S1: Sample descriptive statistics, by region

|  | | Njombe (*N*=159) | | Tabora (*N*=166) | | All | (*N*=325) |  |
| --- | --- | --- | --- | --- | --- | --- | --- | --- |
|  |  | Count | % | Count | % | Count | % |  |
| Social demographics | | | | | | | |  |
| Location type | Rural | 85 | 53.5% | 81 | 48.8% | 166 | 51.1% |  |
|  | Urban | 74 | 46.5% | 85 | 51.2% | 159 | 48.9% |  |
|  |  |  |  |  |  |  |  |  |
| Religion | Christian | 153 | 96.2% | 85 | 51.2% | 238 | 73.2% |  |
|  | Muslim | 2 | 1.3% | 40 | 24.1% | 42 | 12.9% |  |
|  | Other | 4 | 2.5% | 41 | 24.7% | 45 | 13.8% |  |
|  |  |  |  |  |  |  |  |  |
| Age (median) |  | 159 | 27 years | 166 | 26 years | 325 | 27 years |  |
|  |  |  |  |  |  |  |  |  |
| Ethnicity | Bena | 132 | 83.0% | 0 | 0.0% | 132 | 40.6% |  |
|  | Pangwa | 8 | 5.0% | 0 | 0.0% | 8 | 2.5% |  |
|  | Kinga | 4 | 2.5% | 1 | 0.6% | 5 | 1.5% |  |
|  | Hehe | 7 | 4.4% | 0 | 0.0% | 7 | 2.2% |  |
|  | Nyamwezi | 0 | 0.0% | 60 | 36.1% | 60 | 18.5% |  |
|  | Sukuma | 0 | 0.0% | 92 | 55.4% | 92 | 28.3% |  |
|  | Others | 8 | 5.0% | 13 | 7.8% | 21 | 6.5% |  |
|  |  |  |  |  |  |  |  |  |
| Highest level of education | Never | 2 | 1.3% | 9 | 5.5% | 11 | 3.4% |  |
|  | Incomplete Primary | 12 | 7.6% | 14 | 8.5% | 26 | 8.0% |  |
|  | Complete Primary | 109 | 69.0% | 107 | 64.8% | 216 | 66.9% |  |
|  | Complete Secondary | 31 | 19.6% | 32 | 19.4% | 63 | 19.5% |  |
|  | Diploma | 2 | 1.3% | 0 | 0.0% | 2 | 0.6% |  |
|  | Higher Education | 2 | 1.3% | 1 | 0.6% | 3 | 0.9% |  |
|  | Other | 0 | 0.0% | 2 | 1.2% | 2 | 0.6% |  |
|  |  |  |  |  |  |  |  |  |
| Owns mobile telephone | Yes | 150 | 94.3% | 149 | 89.8% | 299 | 92.0% |  |
|  | No | 9 | 5.7% | 17 | 10.2% | 26 | 8.0% |  |
|  |  |  |  |  |  |  |  |  |
| Owns Land | Yes | 150 | 94.3% | 151 | 91.0% | 301 | 92.6% |  |
|  | No | 9 | 5.7% | 15 | 9.0% | 24 | 7.4% |  |
| Circumcision | | | | | | | |  |
| Circumcised | Yes | 107 | 67.3% | 109 | 65.7% | 216 | 66.5% |  |
|  | No | 52 | 32.7% | 57 | 34.3% | 109 | 33.5% |  |
|  |  |  |  |  |  |  |  |  |
| Type of Circumcision | Medical | 94 | 87.0% | 101 | 92.7% | 195 | 89.9% |  |
|  | Traditional | 14 | 13.0% | 5 | 4.6% | 19 | 8.8% |  |
|  | Don’t Remember | 0 | 0.0% | 3 | 2.8% | 3 | 1.4% |  |
| Sexual behaviour | | | | | | | |  |
| Has had sex in past 12 months | Yes | 132 | 83.0% | 157 | 94.6% | 289 | 88.9% |  |
|  | No | 27 | 17.0% | 8 | 4.8% | 35 | 10.8% |  |
|  | Refusal | 0 | 0.0% | 1 | 0.6% | 1 | 0.3% |  |
|  |  |  |  |  |  |  |  |  |
| Number of sex partners in past 12 months | 1 | 81 | 61.4% | 77 | 48.7% | 158 | 54.5% |  |
|  | 2 | 28 | 21.2% | 37 | 23.4% | 65 | 22.4% |  |
|  | 3 | 15 | 11.4% | 25 | 15.8% | 40 | 13.8% |  |
|  | 4 | 2 | 1.5% | 7 | 4.4% | 9 | 3.1% |  |
|  | 5 | 3 | 2.3% | 6 | 3.8% | 9 | 3.1% |  |
|  | 6 | 2 | 1.5% | 3 | 1.9% | 5 | 1.7% |  |
|  | 7 | 1 | 0.8% | 0 | 0.0% | 1 | 0.3% |  |
|  | 19 | 0 | 0.0% | 1 | 0.6% | 1 | 0.3% |  |
|  | 20 | 0 | 0.0% | 1 | 0.6% | 1 | 0.3% |  |
|  | Refusal | 0 | 0.0% | 1 | 0.6% | 1 | 0.3% |  |
|  |  |  |  |  |  |  |  |  |
| Condom use last sex | Yes | 68 | 50.4% | 61 | 38.6% | 129 | 44.0% |  |
|  | No | 67 | 49.6% | 96 | 60.8% | 163 | 55.6% |  |
|  | Refusal | 0 | 0.0% | 1 | 0.6% | 1 | 0.3% |  |
| Knowledge and attitudes | | | | | | | |  |
| VMMC protects 100% against HIV | Yes | 63 | 39.6% | 83 | 50.0% | 146 | 44.9% |  |
|  | No | 81 | 50.9% | 71 | 42.8% | 152 | 46.8% |  |
|  | Don’t know | 15 | 9.4% | 12 | 7.2% | 27 | 8.3% |  |
|  | Refusal | 0 | 0.0% | 0 | 0.0% | 0 | 0.0% |  |
|  |  |  |  |  |  |  |  |  |
| VMMC protects women against cervical cancer | Yes | 95 | 60.1% | 94 | 56.6% | 189 | 58.3% |  |
|  | No | 22 | 13.9% | 25 | 15.1% | 47 | 14.5% |  |
|  | Don’t know | 41 | 25.9% | 47 | 28.3% | 88 | 27.2% |  |
|  | Refusal | 0 | 0.0% | 0 | 0.0% | 0 | 0.0% |  |
|  |  |  |  |  |  |  |  |  |
| VMMC makes sex enjoyable | Yes | 112 | 71.8% | 122 | 73.5% | 234 | 72.7% |  |
|  | No | 18 | 11.5% | 22 | 13.3% | 40 | 12.4% |  |
|  | Don’t know | 26 | 16.7% | 21 | 12.7% | 47 | 14.6% |  |
|  | Refusal | 0 | 0.0% | 1 | 0.6% | 1 | 0.3% |  |
|  |  |  |  |  |  |  |  |  |
| Only men with multiple partners need VMMC | Yes | 12 | 7.5% | 13 | 7.8% | 25 | 7.7% |  |
|  | No | 138 | 86.8% | 141 | 84.9% | 279 | 85.8% |  |
|  | Don’t know | 9 | 5.7% | 12 | 7.2% | 21 | 6.5% |  |
|  | Refusal | 0 | 0.0% | 0 | 0.0% | 0 | 0.0% |  |
