# Supplementary material for: Using discrete choice experiments to inform the design of complex interventions
Source: Trials. 2019 Mar 4;20:157. doi: 10.1186/s13063-019-3186-x (PMC6399844; doi:10.1186/s13063-019-3186-x)
Supplement: Supplementary file 3 — Comparison of estimators. Table S2. Full model estimates. Figure S1 Comparison of main effects by estimator (DOCX 164 kb) [file 13063_2019_3186_MOESM3_ESM.docx]

# Additional file 3: Comparison of estimators

This table provides an overview of the estimated utilities across estimators and including the full output and model statistics. The coefficients represent relative preferences, i.e. utilities. These are effects coded. Reference case utilities are retrieved as: -1*sum(utilities all other attribute levels). For example the utility partner counselling NOT available is -1*(0.354)=-0.354) and No age specific services is -1*(0.037+0.205)=-0.242. This holds for Table A2 as well as Figure A1. Though the GMXL model has the lowest log likelihood ratios and AIC/n values, commonly applied diagnostics used to identify the best fitting model, the lack of significance on the Gamma and Sigma coefficients indicate the absence of both scale heterogeneity and unobserved heterogeneity. This leads us to reject the GMXL and a potential random parameters logit (RPL) models, in favour of the MNL model with interactions. We should note however, as mentioned above that both models provide consistent relative preferences thus in practice will generate the largely same recommendations for intervention design. Uptake predictions based on these models may differ. When used in parametrising uptake in cost-effectiveness models, such as applied by Terris-Prestholt et al (2016) and Quaife et al (2018a) and Quaife (2018b), it is recommended to focus on relative uptake rather than as a very precise indication of absolute uptake [1-3].

**Table S2 Full model estimates**

| Dependent variable: Choice |  | **MNL** |  |  |  | **MNLX** |  |  |  | **GMXL** |  |  |
| --- | --- | --- | --- | --- | --- | --- | --- | --- | --- | --- | --- | --- |
|  |  | Coefficient | | *St. Err* |  | Coefficient | | *St. Err* |  | Coefficient | | *St. Err* |
| Out of hours provision (EX_H) |  | -0.002 |  | *0.023* |  | -0.086 |  | *0.023* |  | 0.015 |  | *0.128* |
| Age specific services – parallel by age (OWNA) | | 0.037 |  | *0.034* |  | 0.096 |  | *0.034* |  | 0.124 |  | *0.163* |
| Age specific services – waiting areas by age (SPW) | | 0.205 | *** | *0.038* |  | 0.212 | ** | *0.037* |  | 0.560 | ** | *0.229* |
| HIV testing opt-in (T_IN) |  | 0.153 | *** | *0.040* |  | 0.188 | ** | *0.036* |  | 0.163 |  | *0.166* |
| HIV testing not available (T_NA) |  | -0.290 | *** | *0.051* |  | -0.399 | *** | *0.044* |  | -0.595 | *** | *0.225* |
| Provider Male (PR_M) |  | 0.243 | *** | *0.037* |  | 0.151 | * | *0.036* |  | 0.600 | *** | *0.200* |
| Provider Female (PR_F) |  | -0.307 | *** | *0.036* |  | -0.235 | *** | *0.036* |  | -0.888 | *** | *0.261* |
| Partner Counselling Available (PRTC) | | 0.354 | *** | *0.031* |  | 0.433 | *** | *0.025* |  | 1.031 | *** | *0.295* |
| Incentive lottery |  | -0.123 | *** | *0.044* |  | -0.141 | *** | *0.041* |  | -0.101 | * | *0.055* |
| Incentive transport voucher |  | 0.208 | *** | *0.044* |  | 0.202 | *** | *0.044* |  | 0.262 | *** | *0.055* |
| Incentive expected value |  | 0.027 |  | *0.029* |  | 0.023 |  | *0.030* |  | -0.048 |  | *0.038* |
| NEITHER |  | -1.304 | *** | *0.094* |  | -1.332 | *** | *0.098* |  | -1.232 | *** | *0.079* |
| Neither * circumcised |  | 0.205 | ** | *0.099* |  | 0.242 | ** | *0.103* |  | 0.170 | *** | *0.048* |
| EX_H* Njombe (NJO) | *Heterogeneity in mean* | | | | | 0.069 | *** | *0.024* |  | 0.207 | ** | *0.088* |
| EX_H* 21 years and older (21) |  |  |  |  |  | 0.097 | * | *0.054* |  | 0.080 |  | *0.125* |
| OWNA:NJO |  |  |  |  |  | 0.038 |  | *0.036* |  | 0.134 |  | *0.095* |
| OWNA:21 |  |  |  |  |  | -0.051 |  | *0.076* |  | 0.048 |  | *0.160* |
| SPW:NJO |  |  |  |  |  | 0.039 |  | *0.038* |  | 0.093 |  | *0.103* |
| SPW:21 |  |  |  |  |  | -0.022 |  | *0.085* |  | -0.087 |  | *0.201* |
| T_IN:NJO |  |  |  |  |  | -0.040 |  | *0.036* |  | -0.143 |  | *0.101* |
| T_IN:21 |  |  |  |  |  | -0.032 |  | *0.080* |  | 0.069 |  | *0.163* |
| T_NA:NJO |  |  |  |  |  | 0.012 |  | *0.045* |  | 0.029 |  | *0.107* |
| T_NA:21 |  |  |  |  |  | 0.100 |  | *0.101* |  | 0.037 |  | *0.195* |
| PR_M:NJO |  |  |  |  |  | 0.087 | ** | *0.037* |  | 0.169 |  | *0.106* |
| PR_M:21 |  |  |  |  |  | 0.125 |  | *0.080* |  | 0.268 | * | *0.158* |
| PR_F:NJO |  |  |  |  |  | -0.086 | ** | *0.037* |  | -0.241 | ** | *0.104* |
| PR_F:21 |  |  |  |  |  | -0.097 |  | *0.077* |  | -0.018 |  | *0.180* |
| PRTC:NJO |  |  |  |  |  | -0.053 | ** | *0.026* |  | -0.176 | * | *0.105* |
| PRTC:21 |  |  |  |  |  | -0.093 |  | *0.057* |  | -0.213 |  | *0.205* |
| NsEX_H |  | *Distns. Of RPs . Std. Devs* | | | | |  |  |  | 0.336 | *** | *0.118* |
| NsOWNAGE |  |  |  |  |  |  |  |  | | 0.227 |  | *0.189* |
| NsSPW |  |  |  |  |  |  |  |  | | 0.519 | *** | *0.159* |
| NsT_IN |  |  |  |  |  |  |  |  |  | 0.470 | *** | *0.178* |
| NsT_NA |  |  |  |  |  |  |  |  |  | 0.478 | ** | *0.192* |
| NsPR_M |  |  |  |  |  |  |  |  |  | 0.456 | *** | *0.143* |
| NsPR_F |  |  |  |  |  |  |  |  |  | 0.259 |  | *0.181* |
| NsPRTC |  |  |  |  |  |  |  |  |  | 1.107 | *** | *0.241* |
| TauScale |  | *Scale parameter* | | | | | |  |  | 0.427 |  | *5.004* |
| GammaMXL |  | *Weighting parameter* | | | | | |  |  | - |  | *-* |
| Sigma(i) |  | *Sample Mean* | | |  |  |  |  |  | 0.221 |  | *0* |
| ll |  | -2,643.21 | |  |  | -2,435.10 | |  | | -2,542.63 | |  |
| AIC/N |  | 1.817 |  |  |  | 1.814 |  |  |  |  | 1.77 |  |
| N= |  | 2,924 |  |  |  | 2,924 |  |  |  |  | 2924 |  |

St.Err: standard errors. The significance levels of the utilities test if the utilities are significantly different from the utility of the reference (i.e. the omitted) category. * p-value<0.1; ** p-value<0.05; *** p-value<0.01.

Figure S1 Comparison of main effects by estimator: The horizontal axis represents relative strength of preferences, with negative values being *relatively* disliked and positive values relatively preferred. The three bars each represent a different estimation approach, with the dark red bars being the simplest multinomial logit (MNL) model without variation in preferences by age and region, the striped bars allow for preference heterogeneity (MNLX) by observed characteristics (age and region) and the while bars additionally allows for unobserved preference and scale heterogeneity (GMXL). It can be see that, though the magnitude of the utility values vary, the relative strength of preferences across attributes are largely robust to estimator.

The significance levels of the utilities test if the utilities are significantly different from the utility of the reference (i.e. the omitted) category. * p-value<0.1; ** p-value<0.05; *** p-value<0.01.

1. Terris-Prestholt F, Quaife M, Vickerman P: **Parameterising User Uptake in Economic Evaluations: The role of discrete choice experiments**. *Health Econ* 2016, **25 Suppl 1**:116-123.

2. Quaife M, Terris-Prestholt F, Eakle R, Cabrera Escobar MA, Kilbourne-Brook M, Mvundura M, Meyer-Rath G, Delany-Moretlwe S, Vickerman P: **The cost-effectiveness of multi-purpose HIV and pregnancy prevention technologies in South Africa**. *J Int AIDS Soc* 2018, **21**(3).

3. Quaife M, Terris-Prestholt F, Di Tanna GL, Vickerman P: **How well do discrete choice experiments predict health choices? A systematic review and meta-analysis of external validity**. *Eur J Health Econ* 2018, **19**(8):1053-1066.
